# Association of Soluble Suppression of Tumorigenesis 2 (sST2) With Platelet Activation, Monocyte Tissue Factor and Ischemic Outcomes Following Angioplasty and Stenting

**DOI:** 10.3389/fcvm.2020.605669

**Published:** 2020-12-22

**Authors:** Stefan Stojkovic, Svitlana Demyanets, Christoph W. Kopp, Christian Hengstenberg, Johann Wojta, Beate Eichelberger, Simon Panzer, Thomas Gremmel

**Affiliations:** ^1^Department of Internal Medicine II, Medical University of Vienna, Vienna, Austria; ^2^Department of Laboratory Medicine, Medical University of Vienna, Vienna, Austria; ^3^Core Facilities, Medical University of Vienna, Vienna, Austria; ^4^Ludwig Boltzmann Institute for Cardiovascular Research, Vienna, Austria; ^5^Department of Blood Group Serology and Transfusion Medicine, Medical University of Vienna, Vienna, Austria; ^6^Department of Internal Medicine I, Cardiology and Intensive Care Medicine, Landesklinikum Mistelbach-Gänserndorf, Mistelbach, Austria

**Keywords:** peripheral artery disease, sST2, ischemic outcomes, platelet reactivity, tissue factor

## Abstract

**Background:** Peripheral artery disease (PAD) patients undergoing infrainguinal angioplasty with stenting suffer high rates of target lesion restenosis and ischemic events. Blood-based prognostic markers in these patients are currently limited. The IL-33/ST2-system is involved in atherothrombosis. Soluble ST2 has been proposed as a biomarker in patients with cardiovascular disease.

**Aim:** To investigate the association of sST2 with platelet activation and monocyte tissue factor (TF) in 316 patients undergoing elective angioplasty and stenting for cardiovascular disease, and its predictive value for ischemic outcomes following infrainguinal angioplasty with stent implantation in 104 PAD patients within this cohort.

**Methods and Results:** Circulating levels of sST2, platelet surface P-selectin, monocyte TF expression as well as soluble P-selectin were determined in 316 consecutive patients on dual antiplatelet therapy following angioplasty and stenting. sST2 was independently associated with soluble P-selectin (B = 6.4, 95% CI 2.0–10.7, *p* = 0.004) and TF expression (B = 0.56, 95% CI 0.02–1.1, *p* = 0.041) but not with platelet surface P-selectin (B = 0.1, 95% CI −0.1–0.3, *p* = 0.307) after adjustment for age, sex, clinical risk factors and inflammatory parameters. During the follow-up of 24 months, the primary endpoint occurred in 41 of 104 PAD patients (39.4%). However, circulating levels of sST2 did not predict the primary endpoint in PAD patients (HR 1.1, 95% CI 0.76–1.71, *p* = 0.527).

**Conclusion:** sST2 is associated with soluble P-selectin and monocyte TF expression in atherosclerosis but not with ischemic outcomes following infrainguinal angioplasty with stent implantation for PAD.

## Introduction

Peripheral artery disease (PAD) remains a great therapeutic challenge with angioplasty and stent implantation being an effective therapeutic intervention in patients with high-grade stenosis (1, 2). However, target vessel restenosis and ischemic events are frequent and severe complications after infrainguinal endovascular interventions. Blood-based biomarkers allowing risk stratification of patients with PAD following angioplasty and stenting are limited (3, 4).

The main components of the interleukin (IL)-33/suppression of tumorigenesis-2 (ST2) system include the ligand IL-33 as well as transmembrane ST2 [ST2L or IL-1 Receptor Like 1 (IL1RL1)] and soluble ST2 (sST2) representing two receptor isoforms (5). sST2 acts as a decoy receptor through binding free IL-33, which prevents cytokine signaling via ST2L (6).

Association of IL-33 and sST2 with the pathogenesis of atherosclerosis and thrombosis has been demonstrated in several previous studies (7–10). In monocytes, the IL-33/ST2 system induces tissue factor (TF) expression and the release of prothrombotic microvesicles (9). Increased levels of IL-33 are associated with an increased risk of in-stent restenosis and sST2 was associated with disease severity and outcome in patients with coronary artery disease (11, 12). Very high IL-33 levels predicted mortality in STEMI patients (11). Both IL-33 and sST2 are elevated in patients with carotid artery stenosis, and correlated with the vulnerability of atherosclerotic plaques (13). The biomarker potential of sST2 was proven in patients with acute myocardial infarction (AMI) (11, 14, 15), heart failure (HF) (16, 17), and critically ill patients (18). In detail, higher sST2 levels were associated with poor outcomes. In contrast, circulating sST2 levels did not predict future cardiovascular events in patients with carotid artery stenosis over a follow-up of 3 years (19). Therefore, the predictive value of circulating sST2 for ischemic outcomes seems to be dependent among others on the localization and manifestation of atherosclerosis.

Data on sST2 in PAD are limited. A previous study demonstrated higher levels of sST2 in patients with PAD compared with healthy controls (20). Another study investigated the influence of IL1RL1 single nucleotide polymorphisms on sST2 levels in PAD patients and found lower sST2 levels in rs950880 AA homozygotes (21). The authors subsequently demonstrated that the combination of a high sST2 level and rs950880 AA homozygosity was a strong predictor of all-cause death, but not for secondary endpoints defined as cardiovascular death, AMI, hospitalization for HF, stroke, and amputation in PAD patients (21). Therefore, the predictive value of circulating sST2 for target vessel restenosis in PAD is still unclear. Moreover, data linking sST2 with platelet activation in atherosclerosis are scarce. We therefore sought to investigate the association of sST2 with platelet activation and monocyte tissue factor (TF) expression in 316 patients undergoing elective angioplasty and stenting for cardiovascular disease, and its ability to predict ischemic outcomes following infrainguinal angioplasty with stent implantation in 104 PAD patients within this cohort.

## Methods

### Study Population

**Figure 1:**
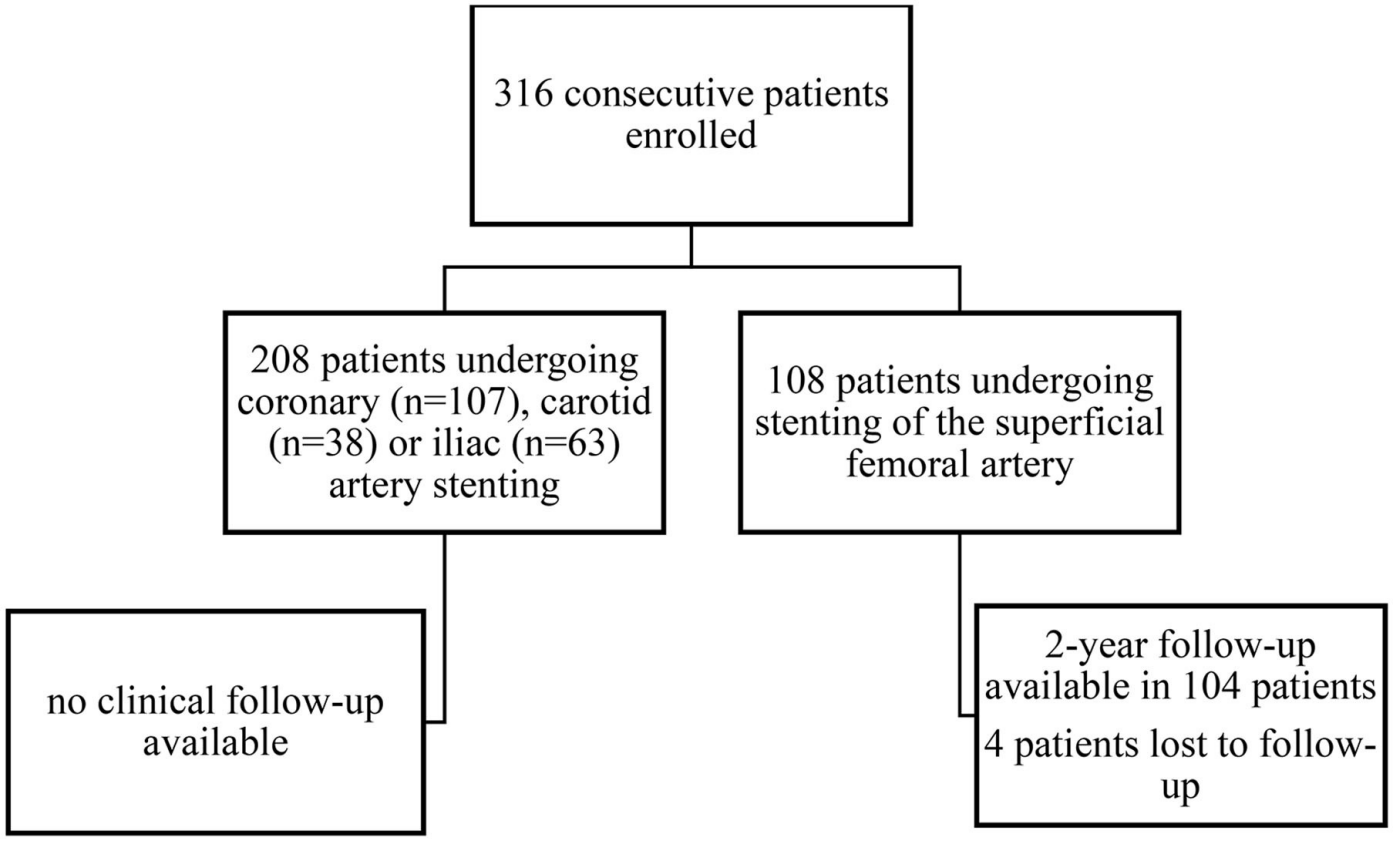
Study design and procedural flow chart.

In this prospective cohort study, 316 consecutive patients undergoing successful angioplasty with endovascular stent implantation were enrolled at the Department of Internal Medicine II at the Medical University of Vienna. Study design, patient enrollment and follow-up are depicted in Figure 1. All patients received 100 mg of aspirin and 75 mg of clopidogrel per day. Patients undergoing peripheral or carotid angioplasty and stenting received DAPT for 3 months followed by aspirin monotherapy. Patients undergoing coronary angioplasty and stenting received DAPT for 6 months, followed by aspirin monotherapy. In 104 patients undergoing infrainguinal angioplasty and stenting clinical follow-up was assessed 1 and 2 years after the endovascular intervention.

Exclusion criteria were a known aspirin or clopidogrel intolerance (allergic reactions, gastrointestinal bleeding), a therapy with vitamin K antagonists (warfarin, phenprocoumon, acenocoumarol) or direct oral anticoagulants (dabigatran, rivaroxaban, apixaban, edoxaban), a treatment with ticlopidine, dipyridamole or non-steroidal anti-inflammatory drugs, a family or personal history of bleeding disorders, malignant paraproteinemias, myeloproliferative disorders or heparin-induced thrombocytopenia, severe hepatic failure, acute and chronic inflammatory diseases, known qualitative defects in thrombocyte function, a major surgical procedure within 1 week before enrolment, a platelet count <100,000 or >450,000/L and a hematocrit <30% as previously described (22, 23).

The study protocol was approved by the Ethics Committee of the Medical University of Vienna in accordance with the Declaration of Helsinki and written informed consent was obtained from all study participants.

### Blood Sampling

Blood was drawn for flow cytometry 1 day after the percutaneous intervention as previously described (24). Flow cytometry was performed by a single operator, blinded to clinical follow-up and sST2 measurements.

### Quantification of sST2

Circulating sST2 was assessed using human ST2/IL-1 R4 DuoSet® ELISA Kit (R&D Systems), as previously described (11, 17, 18, 25).

### Soluble P-Selectin (sP-Selectin)

sP-selectin was quantified in duplicates using 100 μL platelet-poor plasma diluted 20-fold in sample diluent using Human sP-selectin/CD62P ELISA reagent set (R&D Systems), as previously described (26, 27).

### Platelet Surface Expression of P-Selectin

The expression of P-selectin was determined in citrate-anticoagulated blood, as previously described (28, 29). In brief, whole blood was diluted in phosphate-buffered saline to obtain 20 × 10^3^ platelets/μL in 20μL, and incubated with the platelet specific monoclonal antibody anti-CD42b (clone HIP1, allophycocyanin labelled; Becton Dickinson (BD), San Jose, CA, USA). Samples were then incubated with an antibody against P-selectin (anti-CD62p-phycoerythrin, clone CLB-Thromb6; Immunotech, Beckman Coulter, Fullerton, CA, USA). After 15min of incubation in the dark, the reaction was stopped by adding 500 μL PBS and samples were acquired immediately on a FACS Calibur flow cytometer (BD) with excitation by an argon laser at 488 nm and a red diode laser at 635 nm at a rate of 200–600 events per second. Isotype-matched control antibodies were used in separate vials for the determination of non-specific binding. At acquisition, the platelet population was identified by its characteristics in the forward scatter versus side scatter plot. A total of 10,000 events were acquired within this gate. This population was further identified by platelets stained with the platelet-specific monoclonal antibody anti-CD42b versus side scatter. Binding of the antibody against P-selectin was determined in a histogram for P-selectin. The MFI based on all events was used for statistical calculations. The gated events were analyzed using the CellQuest Pro software (BD). Standard BD Calibrite beads were used for daily calibration of the cytometer.

### Monocyte TF

Monocyte TF was measured as previously described (30), using fluorochrome-conjugated monoclonal antibodies: APC-labeled monoclonal antibody for the constitutive platelet marker CD42b (glycoprotein Ib of von Willebrand factor receptor complex), a FITC-labeled monoclonal antibody for human TF (BD, Franklin Lakes, NJ, USA), a PE-Cy5–labeled monoclonal antibody for monocyte CD14 (endotoxin receptor), and corresponding isotype controls. All antibodies were purchased from BD, except the anti-TF monoclonal antibody which came from American Diagnostica (Stamford, CT, USA). In brief, 100 μL of citrate-anti-coagulated whole blood was stained with saturating concentrations of the above mentioned fluorochrome-conjugated monoclonal antibodies. After 10 min of pre-incubation with antibodies in the dark at room temperature, samples were fixed and erythrolysed with Optilyse B (Instrumentation Laboratories, Bedford, MA, USA). Flow cytometry was performed on a FACSCalibur BD. Acquisition was stopped when 5,000 CD14^+^ events were acquired. Monocytes were identified by gating CD14^+^ events, and all additional analyses were performed on this population. The negative and positive delineators were set by gating ~2% background staining on the isotype control fluorescence. TF expression was calculated as percentage of monocytes staining positive for TF (TF^+^-monocytes).

### Clinical Endpoints

Clinical follow-up was assessed at regular visits of the study participants to the outpatient department of the Division of Vascular Medicine at the Medical University of Vienna and via telephone calls, respectively. The primary endpoint was defined as the composite of the first occurrence of any of the following events: non-fatal AMI, non-fatal stroke or transient ischemic attack (TIA), cardiovascular death, and sonographically confirmed >80% target vessel restenosis or reocclusion within 2 years after peripheral angioplasty and stenting.

### Statistical Analysis

Categorical variables are summarized as counts and percentages and are compared by the χ^2^-test or the Fisher's exact test as appropriate. Continuous variables are expressed as median and interquartile range (IQR) and compared by the *t*-test or the Mann-Whitney *U*-test in case of non-normal distribution. Univariate and multivariate linear regression models were fit to evaluate the associations of sST2 with platelet surface P-selectin, sP-selectin, and monocyte TF expression. The multivariate linear regression model was adjusted for age, sex, active smoking, type 2 diabetes, hypertension, hyperlipidemia and high sensitivity C-reactive protein (hs-CRP). Forest plots were plotted using Beta values and 95% CI from linear regression models. The univariate Cox proportional hazard regression model was fit to assess whether sST2 could significantly predict the dichotomous clinical outcome (without/with adverse ischemic events). Hazard ratios (HR) are given as HR per one increase of standard deviation (HR per 1-SD). Kaplan-Meier failure plots were constructed in groups according to sST2 expression above or below the median value to compare time-dependent discriminative power of circulating sST2. Two-sided *p*-values of 0.05 indicated statistical significance. SPSS 22.0 (IBM Corporation, Armonk, NY, USA), GraphPad Prism 8.0 (GraphPad Software Inc., San Diego, CA, USA) and STATA version 12 (StataCorp LLC, College Station, TX, USA) were used for all statistical analyses.

## Results

Clinical characteristics of the overall patient population are given in Table 1. Median age was 66 years (IQR 58–75), and 65.2% of patients were male. Most common comorbidities were hyperlipidemia (93%) and hypertension (90%), followed by active smoking (42%), previous MI (40.5%) and type 2 diabetes (32%).

**Table 1:** Baseline clinical characteristics of the patient population.

	**Overall (*n* = 316)**
**DEMOGRAPHICS**	
Age	66 (58–75)
Male sex	206 (65.2)
**COMORBIDITIES**	
BMI	26.8 (24.2–29.7)
Active smoking	133 (42.1)
Hyperlipidemia	294 (93)
Hypertension	284 (89.9)
Previous MI	128 (40.5)
Previous TIA/stroke	35 (11)
Type 2 diabetes	102 (32.3)
**PRIOR MEDICATION**	
ASA	316 (100)
Clopidogrel	316 (100)
Statin	301 (95.3)
ACE-inhibitor	193 (61.1)
Beta blockers	217 (68.7)
CCB	96 (30.4)
Diuretics	110 (34.8)
**INTERVENTION**	
Coronary artery stent	107 (33.9)
Peripheral artery stent	171 (54.1)
Carotid artery stent	38 (12.0)
DES	107 (33.9)
BMS	206 (65.2)
Genous	3 (0.9)
**LABORATORY PARAMETERS**	
Triglycerides	143 (108–195)
Cholesterol	170 (146–207)
LDL	94 (69–122)
HDL	43 (37-51)
Lipoprotein a	33 (16-80)
hs-CRP	0.79 (0.33–1.81)
Serum creatinine	1.0 (0.9–1.2)
sST2 in pg/mL	1337.9 (1005.0–2022.9)

*BMI, body mass index; MI, myocardial infarction; TIA, transient ischemic attack; ASA, acetylsalicylic acid; ACE, angiotensin converting enzyme; CCB, calcium channel blocker; DES, drug-eluting stent; BMS: bare metal stent; LDL, low-density lipoprotein; HDL, high-density lipoprotein; hs-CRP, high-sensitivity C-reactive protein; sST2, soluble ST2*.

**Figure 2:**
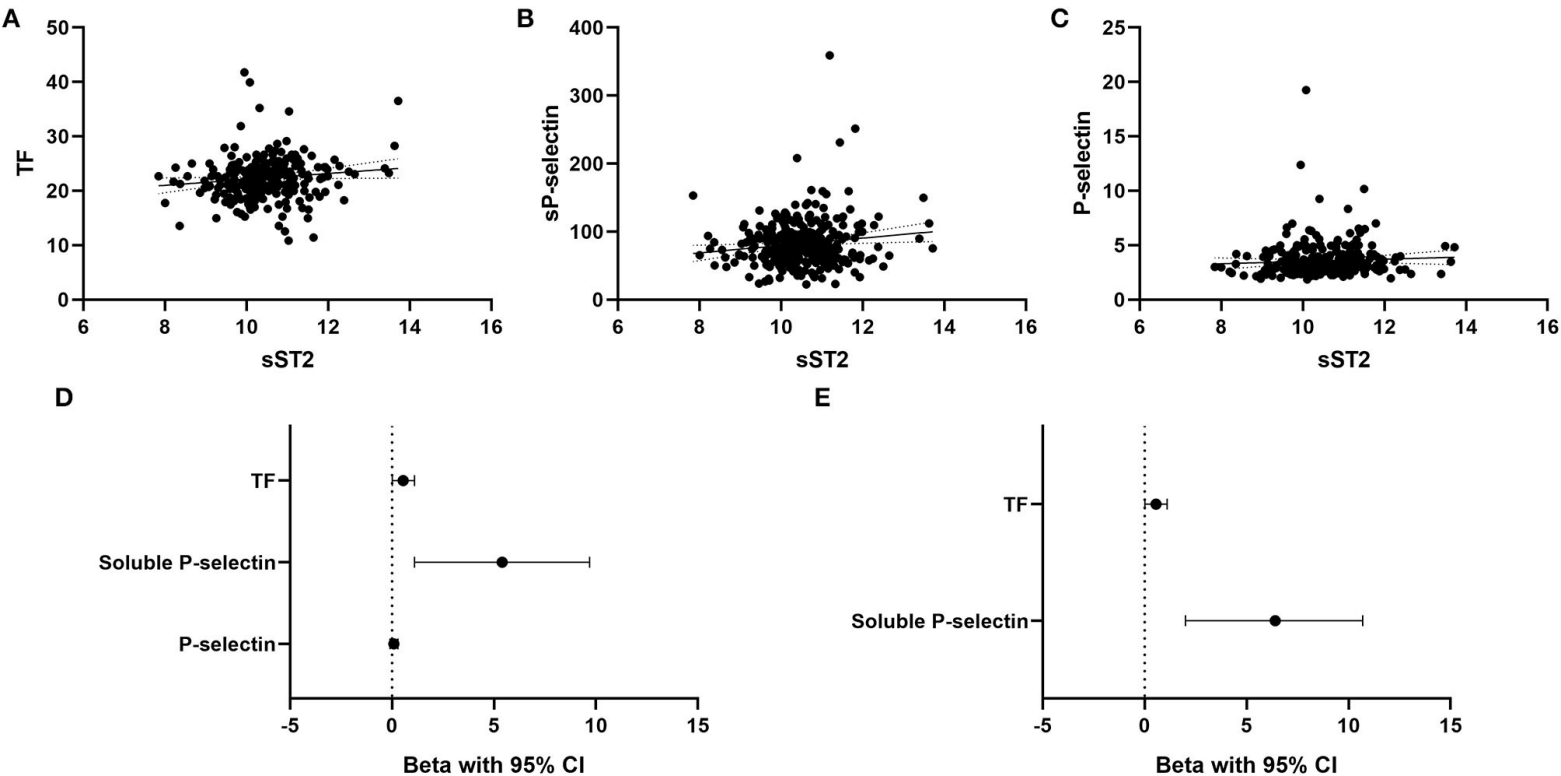
Association of circulating sST2 with monocyte TF, soluble P-selectin, and platelet surface P-selectin. Univariate linear regression analysis for association of sST2 with monocyte TF **(A)**, soluble P-selectin **(B)**, and platelet surface P-selectin **(C)** was performed as described in the methods section. Forest plots depict the univariate **(D)** and multivariate **(E)** regression coefficients Beta with 95% confidence interval. The multivariate regression model was adjusted for age, sex, arterial hypertension, hyperlipidemia, active smoking, type 2 diabetes and high-sensitivity CRP. A *p* < 0.05 was considered statistically significant.

In the overall patient cohort (*n* = 316), sST2 was associated with monocyte TF expression (B = 0.55, 95% CI 0.02–1.1, *p* = 0.042, Figure 2A). Furthermore, sST2 was significantly linked to sP-selectin expression (B = 5.4, 95% CI 1.1–9.7, *p* = 0.014, Figure 2B) but not to platelet surface P-selectin (B = 0.1, 95% CI −0.1–0.3, *p* = 0.307, Figure 2C). Figure 2D depicts respective univariate linear regression coefficient Beta with 95% confidence interval. In the multivariate regression model, sST2 remained significantly associated with sP-selectin (B = 6.4, 95% CI 2.0–10.7, *p* = 0.004, Figure 2E) and monocyte TF expression (B = 0.56, 95% CI 0.02–1.1, *p* = 0.041, Figure 2E) after adjustment for age, sex, clinical risk factors and inflammatory parameters.

**Figure 3:**
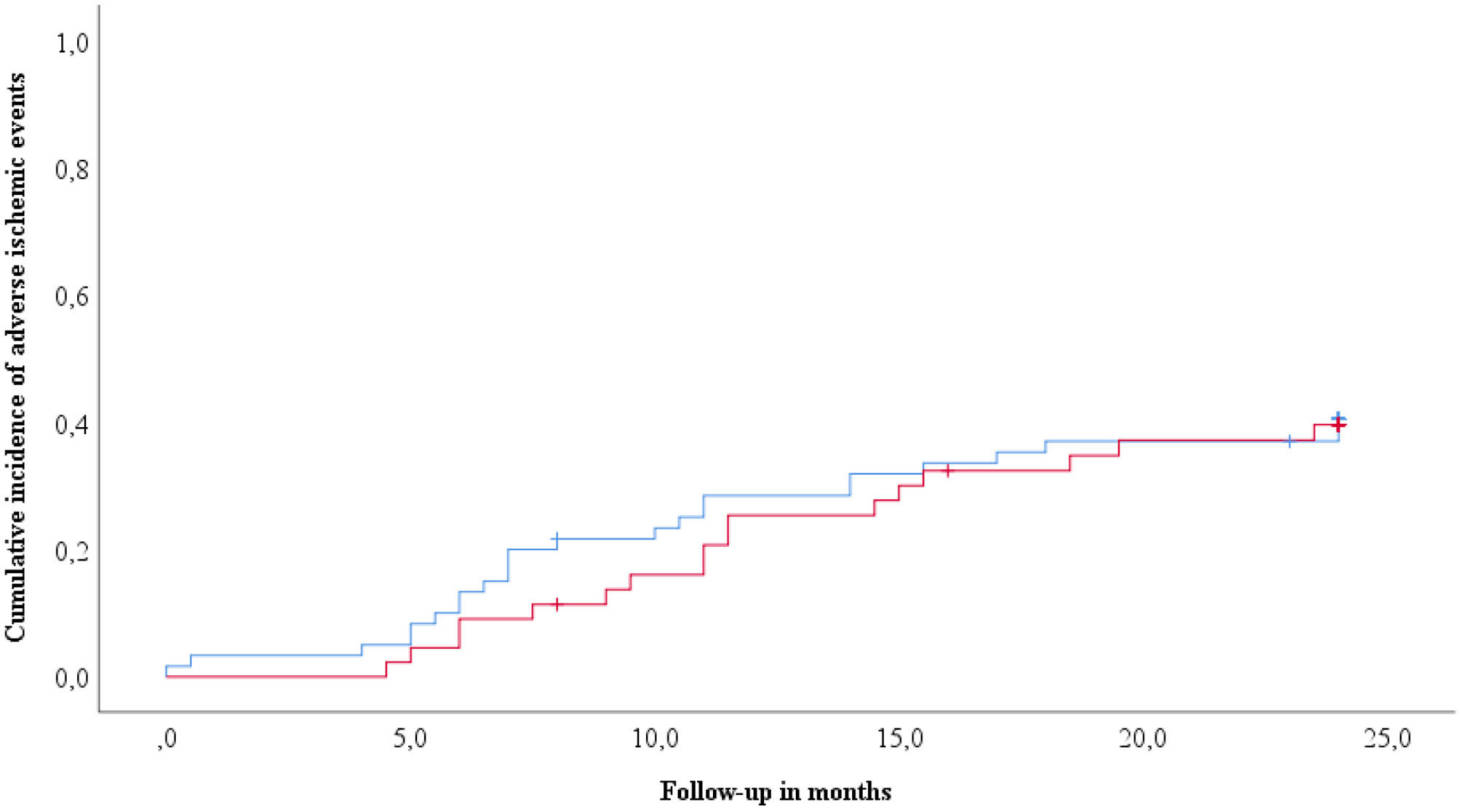
Cumulative incidence of adverse ischemic events according to circulating sST2. Kaplan-Meyer analyses for the cumulative incidence of adverse ischemic events (primary endpoint) following infrainguinal angioplasty and stenting stratified according to circulating sST2 levels. sST2 above the median is indicated by the red line. The blue line indicates sST2 below median.

Two year follow-up was assessed in 104 patients (32.9% of the overall cohort) undergoing elective infrainguinal angioplasty and stenting for PAD. During this time, the primary endpoint occurred in 41 patients (39.4%). Clinical risk factors were similarly distributed between patients without and with the primary endpoint (Table 2). Interestingly, patients suffering the primary endpoint had lower baseline cholesterol and triglyceride levels as compared to patients without the primary endpoint during follow-up. Both groups had similarly high circulating levels of sST2 (1,214 pg/mL, IQR 884–1,775 vs. 1,165 pg/mL, IQR 893–1,838, *p* = 0.976, Table 2). Circulating levels of sST2 did not predict the primary endpoint in Cox regression analysis (HR 1.1, 95% CI 0.76–1.71, *p* = 0.527). Kaplan-Meier survival curves showed no difference in survival for patients with high and low levels of sST2 (log-rank *p* = 0.785, Figure 3).

**Table 2:** Baseline clinical characteristics of the patient population with available follow-up.

	**Overall (*n* = 104)**	**Primary endpoint (*n* = 41)**	**No primary endpoint (*n* = 63)**	***p*-value**
**DEMOGRAPHICS**				
Age	65 (58–72.7)	66 (58.5–75)	63 (58–70)	0.142
Male sex	64 (61.5)	22 (53.7)	42 (66.7)	0.183
**COMORBIDITIES**				
BMI	26.8 (24.5–29.2)	25.6 (24.3–28.5)	27.6 (24.8–29.7)	0.232
Active smoking	46 (44.2)	15 (36.6)	31 (49.2)	0.205
Hyperlipidemia	96 (92.3)	35 (85.4)	61 (96.8)	0.040
Hypertension	96 (92.3)	38 (92.7)	58 (92.1)	0.908
Previous MI	20 (19.2)	6 (14.6)	14 (22.2)	0.337
Previous TIA/stroke	12 (11.4)	3 (7.3)	9 (14.2)	0.129
Type 2 diabetes	38 (36.5)	19 (46.3)	19 (30.2)	0.094
**PRIOR MEDICATION**				
ASA	104 (100)	41 (100)	63 (100)	1
Clopidogrel	104 (100)	41 (100)	63 (100)	1
Statin	94 (90.4)	35 (85.4)	59 (93.7)	0.161
ACE-inhibitor	58 (55.8)	21 (51.2)	37 (58.7)	0.451
Beta blockers	61 (58.7)	24 (58.5)	37 (58.7)	0.984
CCB	40 (38.5)	19 (46.3)	21 (33.3)	0.183
Diuretics	39 (37.5)	13 (31.7)	26 (41.3)	0.325
**LABORATORY PARAMETERS**				
Triglycerides	146.5 (111–211)	125 (102.5–178)	169 (128–241)	0.002
Cholesterol	183 (147.5–222)	168 (144–189.5)	203 (151–239)	0.011
LDL	97 (69–130.8)	93.2 (67.5–118)	108.7 (69.3–153.6)	0.113
HDL	46 (40–54)	47 (42–54)	46 (39−53.5)	0.436
Lipoprotein a	39.5 (16–95.2)	48 (16-101)	38 (16–92)	0.521
hs-CRP	0.9 (0.3–1.7)	0.8 (0.3–1.6)	1.1 (0.5–1.7)	0.274
Serum creatinine	1.0 (0.9–1.2)	1.0 (0.9–1.2)	1.0 (0.9–1.1)	0.695
sST2 in pg/mL	1,213 (890–1,837)	1,214 (884–1,775)	1,165 (893–1,838)	0.976

*BMI, body mass index; MI, myocardial infarction; TIA, transient ischemic attack; ASA, acetylsalicylic acid; ACE, angiotensin converting enzyme; CCB, calcium channel blockers; LDL, low-density lipoprotein; HDL, high-density lipoprotein; hs-CRP, high-sensitivity C-reactive protein; sST2, soluble ST2*.

## Discussion

In the present study we investigated the association of sST2 with platelet activation and monocyte TF expression after elective angioplasty and stenting for cardiovascular disease, and its prognostic value following infrainguinal angioplasty with stent implantation in PAD. We could show here for the first time that sST2 is independently associated with increased levels of sP-selectin and enhanced monocyte TF expression. However, the association of sST2 with a prothrombotic milieu did not translate into a higher risk of adverse ischemic events in patients undergoing infrainguinal angioplasty and stenting for PAD.

Conflicting results have been reported on the role of the IL-33/ST2 system in different inflammatory conditions including atherosclerosis (7–10, 31, 32). While our group previously demonstrated pro-inflammatory and pro-angiogenic effects of IL-33 in human vascular endothelial cells (7, 10) as well as pro-thrombotic effects of IL-33 in human monocytes and endothelial cells (8, 9), another study reported that the injection of IL-33 in atherosclerosis-prone apolipoprotein E (ApoE) knockout (ApoE^−/−^) mice reduced the plaque area via induction of IL-5 and antioxidized low-density lipoprotein (ox-LDL) antibodies (33). However, the knockout of ST2 or IL-33 showed no beneficial effect on atherosclerosis development in ApoE^−/−^ mice (34).

Different blood cells including monocytes, eosinophils, basophils, mast cells, B-cells, innate lymphoid cells type 2, different subtypes of T-cells, dendritic cells, and M2 polarized macrophages express ST2L (31). Platelets on the other hand, do not express ST2L on their surface to our best knowledge (unpublished correspondence). In the present study, we found a strong association of circulating sST2 with monocyte TF expression and sP-selectin but not with platelet surface P-selectin. Platelets shed P-selectin from their surface, which is then detectable in the circulation in its soluble form. Therefore, sP-selectin may be a more stable marker reflecting continuously ongoing platelet activation, while platelet surface P-selectin may represent platelet activation at the time of blood sampling (26). It was previously shown that P-selectin expression on activated platelets as well as sP-selectin are major determinants of leukocyte-platelet interaction and can trigger monocyte TF expression (35, 36). Furthermore, sP-selectin has been repeatedly associated with thromboembolic events (37, 38). We could show previously that IL-33 stimulates increased TF expression in monocytes and the release of prothrombotic extracellular vesicles via ST2L (9). Thus, one could hypothesize that the observed associations of sST2 with increased monocyte TF expression and sP-selectin might reflect complex platelet-monocyte interactions, with increased monocyte/platelet interactions and monocyte TF expression, contributing to a prothrombotic milieu in patients with cardiovascular disease (9).

The association of increased sST2 with adverse outcomes was demonstrated in different cardiovascular pathologies. sST2 concentrations were correlated with short-term as well as long-term mortality in patients with AMI (11, 14). In the large cohort from the Ludwigshafen risk and cardiovascular health study, sST2 predicted all-cause mortality also in patients with stable coronary artery disease (CAD) during a follow-up of 9.8 years (39). Moreover, sST2 is an established biomarker for mortality in patients with acute or chronic HF (16). sST2 independently predicted all-cause mortality in non-ischemic, dilated cardiomyopathy during a follow-up of 7 years, but it was inferior to growth differentiation factor-15 (GDF-15) for the prediction of fatal arrhythmic events in this patient population (17). In children and adolescent with HF sST2 performed poorly in contrast to midregional (MR) pro-atrial natriuretic peptide (proANP) that could accurately detect HF with diagnostic performance comparable with NT-proBNP. However, NT-proBNP, sST2, MR-proANP, and GDF-15 were able to distinguish between pediatric HF patients with preserved and poor functional status (40).

In spite of the observed association of sST2 with increased sP-selectin and monocyte TF expression, the present study showed that sST2 is not capable to identify PAD patients at higher risk for poor outcomes following angioplasty and stenting. Our findings are in line with a previous study by Lin et al., who demonstrated that in patients with PAD, increased sST2 levels were an independent predictor of all-cause mortality, but did not predict other endpoints including cardiovascular death, MI, stroke, and amputation (21). Both groups of patients, i.e., with and without event during the follow-up had similarly elevated sST2 levels, reflecting high cardiovascular risk and prothrombotic environment characteristic for PAD patients. The vast majority of patients with ischemic outcomes in the present study suffered target vessel restenosis (88%) during follow-up, and only 4.8% of patients experienced an AMI. Thus, sST2 is not associated with target vessel restenosis in PAD. Due to the low number of other ischemic events, we cannot draw conclusions on cardiovascular mortality based on the present study in this group of patients.

The present study had several limitations. Blood samples were acquired 24 h after the intervention and all patients were still on dual antiplatelet therapy at the time of blood sampling. Dual antiplatelet therapy can inhibit platelet P-selectin expression, possibly confounding the association of sST2 with platelet activation (41). Thus, we cannot provide data on the variability of sST2 formation, platelet activation and monocyte TF expression over time. Choosing this time point we sought to investigate whether or not a single post-procedural measurement early after intervention may be used for risk stratification. Another limitation was the small sample size in the follow-up cohort. Due to its small sample size, the results of our study should be confirmed in a larger cohort of patients with PAD. We have only used CD14 antibodies and therefore were not able to identify non-classical monocytes. However, based on previous studies, classical and intermediate monocytes are responsible for the most TF expression and prothrombotic potential of monocytes (9). Finally, currently available ELISA cannot distinguish between free and complexed sST2 (42).

In conclusion, circulating levels of sST2 are associated with sP-selectin and monocyte TF expression in atherosclerosis but not with ischemic outcomes following infrainguinal angioplasty with stent implantation for PAD.

## Data Availability Statement

The raw data supporting the conclusions of this article will be made available by the authors, without undue reservation.

## Ethics Statement

The studies involving human participants were reviewed and approved by Ethikkommission der Medizinischen Universität Wien, Borschkegasse 8b/6, 1090 Vienna, Austria. The patients/participants provided their written informed consent to participate in this study.

## Author Contributions

SS, SD, and TG: conceptualization. CK, SP, BE, and TG: data curation. SS and TG: formal analysis. TG: funding acquisition and project administration. SP and TG: investigation. SS and SP: methodology. JW and CH: resources. SS, SD, and TG: writing—original draft. CK, JW, CH, BE, and SP: writing—review and editing. All authors have accepted responsibility for the entire content of this submitted manuscript and approved submission.

## Conflict of Interest

The authors declare that the research was conducted in the absence of any commercial or financial relationships that could be construed as a potential conflict of interest.

## Abbreviations

ApoE: apolipoprotein
ox-LDL: antioxidized low-density lipoprotein
HF: heart failure
HR: hazard ratio
hs-CRP: high sensitivity CRP
IL-33: interleukin 33
IQR: interquartile range
MFI: mean fluorescence intensity
MI: myocardial infarction
PAD: peripheral artery disease
SD: standard deviation
sP-selectin: soluble P-selectin
sST2: soluble suppression of tumorigenesis 2
TF: tissue factor
TIA: transient ischemic attack.
